# The Potential Use of Targeted Proteomics and Metabolomics for the Identification and Monitoring of Diabetic Kidney Disease

**DOI:** 10.3390/jpm14101054

**Published:** 2024-10-11

**Authors:** Nele Van Roy, Marijn M. Speeckaert

**Affiliations:** 1 Department of Endocrinology, Ghent University Hospital, 9000 Ghent, Belgium; nele.vanroy@ugent.be; 2 Department of Nephrology, Ghent University Hospital, 9000 Ghent, Belgium; 3 Research Foundation-Flanders (FWO), 1000 Brussels, Belgium

**Keywords:** diabetic kidney disease, diabetic nephropathy, multi-omics, proteomics, metabolomics, CKD273

## Abstract

Diabetic kidney disease (DKD) is a prevalent microvascular complication of diabetes mellitus and is associated with a significantly worse prognosis compared to diabetic patients without kidney involvement, other microvascular complications, or non-diabetic chronic kidney disease, due to its higher risk of cardiovascular events, faster progression to end-stage kidney disease, and increased mortality. In clinical practice, diagnosis is based on estimated glomerular filtration rate (eGFR) and albuminuria. However, given the limitations of these diagnostic markers, novel biomarkers must be identified. Omics is a new field of study involving the comprehensive analysis of various types of biological data at the molecular level. In different fields, they have shown promising results in (early) detection of diseases, personalized medicine, therapeutic monitoring, and understanding pathogenesis. DKD is primarily utilized in scientific research and has not yet been implemented in routine clinical practice. The aim of this review is to provide an overview of currently available data on targeted omics. After an extensive literature search, 25 different (panels of) omics were withheld and analyzed. Both serum/plasma and urine proteomics and metabolomics have been described with varying degrees of evidence. For all omics, there is still a relative paucity of data from large, prospective, longitudinal cohorts, presumably because of the heterogeneity of DKD and the lack of patient selection in studies, the complexity of omics technologies, and various practical and ethical considerations (e.g., limited accessibility, cost, and privacy concerns).

## 1. Introduction

Diabetic kidney disease (DKD) is a common and dangerous chronic consequence of diabetes mellitus (DM), affecting 20–50% of patients and being the leading cause of chronic kidney disease (CKD) and end-stage kidney disease (ESKD) [1]. DKD pathophysiology consists of complicated interactions between genetic and environmental variables, as well as hemodynamic, metabolic, inflammatory, and fibrotic processes induced by hyperglycemia and oxidative stress, which result in glomerular and tubular injury [1,2,3,4]. High levels of inflammatory markers such C-reactive protein (CRP), interleukin-6 (IL-6), and tumor necrosis factor-alpha (TNF-α) are linked to disease progression and cardiovascular consequences [5]. Chronic hyperglycemia is a major risk factor for DKD, as it initiates a series of harmful metabolic pathways such as the polyol route, the advanced glycation end-products (AGE) pathway, and the activation of protein kinase C. The pathogenesis of DKD is heavily reliant on oxidative stress and inflammation, which are created by these pathways. Mesangial enlargement, elevated intraglomerular pressure, and glomerular hyperfiltration are the outcomes of persistent hyperglycemia. These alterations eventually result in podocyte damage and glomerular basement membrane (GBM) thickening, which causes glomerulosclerosis and tubulointerstitial fibrillation [3]. DKD typically progresses through stages, starting with early hyperfiltration, characterized by increased glomerular filtration rate (GFR) and kidney hypertrophy without detectable abnormalities in urinary albumin excretion. After that, the disease advances to incipient DKD, which is defined by microalbuminuria (30–300 mg/day), which is an indication of kidney damage that begins early even in cases when GFR is normal or slightly elevated. At this point, structural alterations such as mesangial matrix expansion and GBM thickening begin to take place. When DKD progresses, it moves into the overt stage, which is characterized by a progressive loss in GFR and macroalbuminuria (>300 mg/day). There is a higher risk of hypertension and cardiovascular complications during this phase, which also has more noticeable glomerulosclerosis, tubulointerstitial fibrosis, and arterial hyalinosis [1]. The late stage of DKD is defined by a considerable reduction in GFR (15–29 mL/min/1.73 m^2^) and chronic proteinuria [4]. The final stage of DKD progression is ESKD, which is distinguished by a full loss of kidney function and extensive fibrosis, necessitating dialysis or kidney transplantation to save lives [6]. Fibrotic and inflammatory processes have a crucial role in the pathophysiology of DKD. High levels of growth factors (TGF-β) and inflammatory cytokines (IL-6, TNF-α) can cause renal inflammation and fibrosis. The stimulation of the renin–angiotensin–aldosterone system (RAAS) exacerbates these processes by causing renal vasoconstriction and hypertension, which accelerate kidney destruction [2]. 

Although a kidney biopsy is the most accurate diagnostic method, clinical practice relies on urine albumin levels and estimated glomerular filtration rate (eGFR) for diagnosis and staging, despite challenges in early detection [1,2,7]. Omics technologies, such as proteomics, metabolomics, and genomics, have revealed new details on DKD pathophysiology, notably in inflammation-related pathways. These approaches can detect changes in protein and metabolite profiles that may serve as therapeutic targets [6]. Increased levels of kidney injury molecule-1 (KIM-1) and tumor necrosis factor receptor (TNFR) have been linked to a higher risk of ESKD and poor renal outcomes, underscoring the potential of these biomarkers to enhance the diagnosis and prognosis of DKD [7]. Omics technology has the potential to improve therapeutic monitoring, enable personalized therapy, facilitate early detection, and expand our understanding of DKD. The goal of this study is to provide a comprehensive overview of current advances in proteomics and metabolomics in people with DKD who have either T1DM or T2DM. 

## 2. Materials and Methods

A comprehensive literature search was conducted in both PUBMED and EMBASE in August 2023 to identify relevant studies that investigated the diagnostic and prognostic role of omics in patients with diabetes mellitus. Initially, the following MESH terms were used: “multi-omics”, “omics”, “proteomics”, “metabolomics”, “nephropathy”, “diabetes”, and “kidney”. These were subsequently supplemented by “CKD273” and “biomarkers”. To refine the search results, three filters were applied: publication date (articles published over the last 10 years: 2013–2023), language (English), and study type (randomized control trials). The search results were screened for relevance based on the titles and abstracts. The remaining articles were selected for full-text review. The reference lists of the selected articles were screened for additional articles. After collecting the articles, relevant data were extracted and organized for synthesis.

## 3. Results

### 3.1. Descriptive Analysis

**Figure 1:**
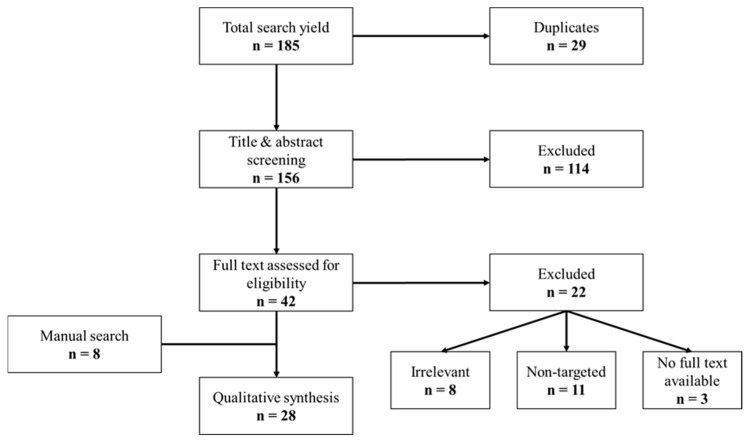
Summary of literature search.

Of the 185 articles, 28 were withheld (Figure 1), describing a total of 25 targeted omics (Table 1). Thirteen were plasma/serum omics, and twelve were urinary omics. A summary of plasma/serum omics can be found in Table 2 and a summary of urinary omics can be found in Table 3. 

Of the 13 different plasma omics groups, 3 belonged to the metabolomics group, 10 to the proteomics group, 11 to the single-omics group, and 2 to the multi-omics group. Of the 12 different urinary omics, 2 belong to the metabolomics group, 10 to the proteomics group, 7 to single-omics, and 5 to multi-omics.

### 3.2. Diabetic Kidney Disease and Urinary Omics

#### 3.2.1. Urinary Proteome/Peptidome and Its Omics

The urinary proteome is a collection of all proteins present in urine and consists of >1500 proteins and >20.000 peptides (short chains of amino acids, the building blocks of proteins). These proteins originate from different sources: kidney-specific proteins (involved in kidney function), plasma-derived proteins (filtered through the glomerulus), and exosomes (membrane-bound vesicles released from cells). The proteome com-position is influenced by numerous internal and external factors such as kidney function, sex, diet, exercise, body composition, age, and disease [33]. Several techniques can be employed when conducting urinary proteomic studies, including mass spectrometry (MS), immunoblotting, enzyme-linked immunosorbent assay (ELISA), nuclear magnetic resonance (NMR), and protein microarrays. Multiple techniques are often used to obtain a comprehensive view of the proteomes. In our review, we found 18 articles concerning 10 different types of urinary proteomics (both single- and multi-omics).

##### CKD273

CKD273 is a capillary electrophoresis–MS-based, multidimensional urinary biomarker, consisting of 273 defined urinary peptides (mainly collagen, albumin, alpha-1-antitrypsin, fibrinogen, …, fragments), identified by Good et al. [34] in 2010 in the context of CKD, irrespective of the underlying pathology. The precise mechanism remains elusive and is currently under investigation [35]. It has shown promise in detecting progressive CKD at an early stage, and therapeutic intervention is more likely to be effective. In DKD, it is the best-known and most extensively researched multi-omic [36]. In this review, eight studies that examined CKD273 in the context of DKD were identified. An overview of the studies concerning CKD273 is provided in Table 4.

##### Diagnostic and Prognostic Biomarker

In normoalbuminuric patients, post hoc analysis of the DIRECT-Protect 2 study [35], the prospective PRIORITY study [37], and the case–control study of Zürbig et al. [18] concluded that the CKD273-classifier was able to predict the early onset of DKD. In the DIRECT-Protect 2 study, progression to microalbuminuria in patients with T2DM was observed with a hazard ratio (HR) of 2.5 (95% CI: 1.4–4.3) in patients with high-risk scores at baseline, as compared to low-risk scores [35]. In patients with T2DM, the PRIORITY trial described progression to microalbuminuria in 28% of patients with high-risk scores, as compared to 9% of patients with low-risk scores, leading to an HR of 2.48 (95% CI: 1.80–3.42). An HR of 5.15 (95% CI: 3.41–7.76) was observed for a 30% decrease in the baseline eGFR in patients with high-risk scores. The HR for doubling in serum creatinine level from baseline was 7.49 (95% CI: 2.97–18.90) [19,37,38]. Zürbig et al. [18] postulated that CKD273 could not only predict progression from normoalbuminuria to microalbuminuria better than the currently used markers but could also predict it sooner. The progression from normo- to microalbuminuria could be predicted 1.5 years earlier than microalbuminuria, and progression from micro- to macroalbuminuria could be predicted 3–5 years before the onset in both patients with T2DM and T1DM.

The results were inconsistent between patients with T2DM and those with microalbuminuria. Currie et al. [20] and Roscioni et al. [21] concluded that CKD273 was associated with (some) renal endpoints. Oellgaard et al. [22] could not show significancy. Although Currie et al. [20] could not demonstrate that high-risk scores of CKD273 were predictive of a 30% eGFR decline, they were able to discriminate patients who transitioned to albuminuria status from those who did not, based on the classifier score. In addition, they showed that CKD273 scores were associated with all-cause mortality independent of established risk factors (primary endpoint). A small sample case–control study by Roscioni et al. [21] showed an association between CKD273 and transition to microalbuminuria or macroalbuminuria (odds ratio of 1.35), suggesting its use as a tool to identify and treat patients at risk of DKD progression. However, in a long-term study with a follow-up of 19 years, Oellgaard et al. [22] could not demonstrate that CKD273 was a predictor of renal endpoints (progression to macroalbuminuria, impaired renal function, and the combination of death and ESKD) in addition to standard clinical variables.

##### Biomarker of Therapeutic Response 

Three study groups assessed the ability of CKD273 to predict therapeutic responses to linagliptin [23], spironolactone [24,37] and candesartan [35]. Linagliptin is a selective dipeptidyl peptidase 4 inhibitor, with glucose-lowering effects, that has also been suggested to exhibit kidney protective effects. Although this could not be concluded from the original MARLINA-T2D trial for the whole cohort, a post hoc analysis of the same trial, performed by Siwy et al. [23], concluded that in patients with high-risk CKD273 scores, there was potential for linagliptin to slow DKD progression. The PRIORITY study was designed to validate CKD273 and assess whether the early initiation of spironolactone, an aldosterone antagonist, could delay the development of microalbuminuria or impair kidney function in individuals with high-CKD273 classifier scores. No significant difference was observed between the placebo and intervention group [37,38]. Inhibition of the renin–angiotensin system is the cornerstone of the management of DKD. The DIRECT trial assessed the treatment effects of candesartan in patients with T1DM and T2DM with normoalbuminuria. The use of candesartan did not prevent the progression of microalbuminuria [39]. It was assumed that better patient selection would result in significant results. However, despite the CKD273 classifier being able to identify normoalbuminuric patients with a high versus low risk of DKD progression, post hoc analysis could not conclude that CKD273 could distinguish responders from non-responders [35].

##### GFR-Based Classifier

CKD273 was developed in a heterogeneous population with CKD. Although it contains proteins and peptides that are clearly associated with the pathogenesis of diabetes, it also includes peptides not relevant to DKD [20]. This issue has been addressed by Oellgaard et al. [22]. They performed a post hoc study of the Steno-2 trial, which not only included CKD273, but also looked at a second GFR-based classifier. This classifier was derived from a subset of ten urinary peptides (mainly collagen fragments) from the CKD273 classifier, which was previously shown to be the most capable of differentiating between CKD progressors and non-progressors in DM. Although this novel classifier showed promising results with regard to renal endpoints, it failed to reach statistical significance (possibly due to the small sample size and smaller than expected hazard ratio). These findings suggest that subsets of the CKD273 classifier may be more useful and worth exploring in the future, as these subsets can be derived from more homogeneous cohort.

##### Urinary Kidney Injury Molecule-1

KIM-1 is a transmembrane glycoprotein that is expressed in the apical membranes of proximal tubular cells. Levels in both plasma and urine increase with tubular injury [30,40]. Concentrations were determined using a particle-enhanced sandwich-type immunoassay based on flow cell fluorometry [13]. In this review, four different studies examined urinary KIM-1 levels in the context of DKD [13,30,31,32,41]. An overview of the studies concerning KIM-1 (both urinary and plasma/serum based) is provided in Table 5.

##### Role in Pathogenesis

In normoalbuminuric patients with T1DM, Nowak et al. [13] showed a weak association between urinary KIM-1 levels and the risk of early progressive renal decline. This association disappeared once the plasma KIM-1 levels were included in the analysis. The researchers described these results as difficult to interpret because there were different explanations for the findings based on the underlying pathophysiology. One possibility is that urinary KIM-1 reflects acute damage, which may vary over time, while plasma KIM-1 concentration reflects the integration of production over time, which is more stable. Another explanation is that both markers reflect different aspects of tubular damage.

In addition, Panduru et al. [31] could not predict progression to microalbuminuria, macroalbuminuria, or ESRD in 1573 patients with T1DM based on urinary KIM-1. However, subsequent multiple regression analysis showed a likely causal association between increased KIM-1 levels and low eGFR. However, the exact underlying mechanism remains unknown.

The post hoc analysis by Dekkers et al. [30] was designed to assess the effect of the sodium–glucose co-transporter (SGLT2) inhibitor dapagliflozin in 33 participants on markers of glomerular and tubular injury and inflammation. All 33 participants had T2DM and had a urine albumin–creatinine ratio (uACR) between 100 and 3500 mg/g creatinine. After six weeks, dapagliflozin appeared to decrease urinary KIM-1 excretion by 22.6%, suggesting an effect of SGLT2 inhibitors on tubular injury (the change in albuminuria correlated with the change in KIM-1 with R = 0.39 and *p* = 0.05). However, this study was not designed to assess the predictive ability of tubular markers for therapeutic response, and the results should be regarded as hypothesis generating rather than hypothesis testing. 

One study [32] confirmed that patients in the highest quartiles of urinary KIM-1 excretion were associated with a rapid decline in kidney function or development of ESKD and could be seen as a predictor of tubulointerstitial damage among patients with T2DM and DKD. An HR of 2.77 (95% CI: 1.27–6.05) was noted.

Previous reviews [13,40] have reported data on urinary KIM-1 as inconclusive, disappointing, and limited. However, despite not consistently showing a significant role for urinary KIM-1 as an early diagnostic marker of DKD or as a predictive therapeutic marker for interventions, urinary KIM-1 seems to play an important role in the pathogenesis of DKD. Further research is needed to unravel the mechanisms of (urinary) KIM-1 in early DKD and/or to evaluate its possible role as a therapeutic target. Plasma KIM-1 will be discussed later.

##### Other

Apart from the urinary proteomics described above, three research groups have described an additional seven single- or multi-omics [15,30,32]. Dekkers et al. [30] not only assessed urinary KIM-1 (e.g., supra) but also examined neutrophil gelatinase-associated lipocalin (NGAL), liver type fatty acid binding protein (LFABP), monocyte chemoattractant protein-1 (MCP-1), and interleukin-6 (IL-6) in relation to dapagliflozin. Apart from the effect on KIM-1, IL-6 excretion decreased by 23.5% after 6 weeks of dapagliflozin treatment compared to placebo. No changes were observed in the other potential biomarkers. The decrease in IL-6 excretion might represent a reduction in intrarenal inflammation secondary to the use of SGLT2 inhibitors. As already mentioned, because of the small sample size and limited power of this study, no clear conclusions can be drawn from the results. However, these findings need to be further confirmed, and additional research is needed to define the exact role of these markers.

The prospective cohort study of Satirapoj et al. [32] was designed to assess the association of tubular damage markers with rapid renal progression and the incidence of ESKD in T2DM at 3-year follow-up. In addition to the previously described KIM-1, they also examined cystatin-C (a marker of renal tubular dysfunction), angiotensinogen (related to the degree of tubulointerstitial damage in DKD), and NGAL (levels of urine NGAL are related to the severity of nephropathy and an increase is seen after acute kidney injury). The highest quartiles of all of the examined markers were associated with rapid decline in kidney function or development of ESKD, with similar (but not better) performance to uACR (area under the curve (AUC) varying from 0.6 to 0.7 and HRs varying between 2.53 and 2.96).

Based on prior research, Colombo et al. [15] selected 13 urine and 9 serum biomarkers to assess whether they could improve the prediction of renal disease progression in T1DM in addition to uACR. Urinary biomarkers examined included the epidermal growth factor/monocyte chemoattractant protein-1 (EGF/MCP-1) ratio, MCP-1, IL-8, EGF, EGF-receptor, IL-18, IL-6, macrophage inflammatory protein-1 β, amphiregulin, placental growth factor, IL-4, epiregulin, and heparin-binding EGF-like growth factor. Among these, in the univariate analysis, only EGF/MCP-1 ratio was associated with kidney disease progression after adjusting for uACR. However, this significance only remained when restricted to those with normo- or microalbuminuria at baseline and when looking at progression to eGFR < 45 mL/min/1.73 m^2^, but not to <30 mL/min/1.73 m^2^. When using a panel of urinary biomarkers (all 13 markers), they could not outperform uACR in predicting the final eGFR.

#### 3.2.2. Urinary Metabolomics

The urinary metabolome refers to the complete set of small-molecule metabolites found in urine. It is a dynamic and complex mixture of end products of various biochemical pathways [11]. They were analyzed using techniques such as nuclear magnetic resonance spectroscopy and mass spectrometry. Five articles on post hoc analysis that described targeted urinary metabolomics were withheld during the literature search [25,26,27,28,29].

##### Role in Pathogenesis

Saulnier et al. [28] performed an exploratory post hoc analysis of the data (including kidney biopsy) of 62 participants from a clinical trial originally designed to examine the efficacy of losartan. They aimed to assess the relationship between 12 metabolites and the kidney structure in DKD. Previous research [42] has identified 13 metabolites with lower urinary concentrations in patients with DKD. However, Saulnier et al. [28] used only 12 compounds because of their presumed relationship with mitochondrial function: 2-ethyl 3-OH propionate, 3-hydroxypropionate, 3-hydroxy isovalerate, 3-methyl-crotonyl glycine, 3-hydroxy isobutyrate, triglyglycine, aconitic acid, citric acid, glycolic acid, homovanillic acid, uracil, and 3-methyl adipic acid. In general, lower concentrations of metabolites correlated with greater (early) structural glomerular lesions (based on morphometric analysis of kidney biopsy specimens), indicating a possible role in pathogenesis and identifying them as potential biomarkers for early DKD. In the cross-sectional Chronic Renal Insufficiency Cohort (CRIC) study, 3-hydroxyisobutyrate and 3-methylcrotonyglycine concentrations had a negative association with the eGFR slope, whereas a positive association was found with citric and aconitic acid. The levels of 3-hydroxyisobutyrate and aconitic acid were associated with higher and lower risk for kidney failure with replacement therapy, respectively (HRs of 2.34 (95% CI: 1.51–3.62) and 0.70 (95% CI: 0.51–0.95)) [29]. 

##### Biomarker of Therapeutic Response

Three studies assessed the predictive ability of urinary metabolomics [25] and its response to treatment [26,27]. Because the response to treatment with spironolactone in patients with DKD has high inter-individual variability, Mulder et al. [25] performed a non-targeted and targeted analysis of urinary metabolomics to identify predictive markers of therapeutic response. Eighteen metabolites were identified in a test cohort of 102 participants using a systems biology and machine learning approach (nontargeted). These could be assigned to the molecular mechanisms of fibrosis, inflammation, and oxidative stress: L-asparagine, L-lysine, 4-hydroxyproline, L-proline, L-aspartic acid, l-glutamic acid, L-arginine, L-cysteine, pyroglutamic acid, l-phenylalanine, l-glutamine, norepinephrine, succinic acid, 2-ketoglutaric acid, l-tryptophan, citric acid, and serotonin. Baseline urinary metabolite scores were calculated for each participant in the targeted replication cohort (targeted). Patients with scores in the lowest tertile of the score showed a higher uACR reduction after treatment with spironolactone (–58%), thus suggesting the use of urinary metabolites as a tool to tailor albuminuria lowering with spironolactone treatment, moving towards personalized medicine.

Atrasentan is a selective inhibitor of the endothelin A receptor that has been shown to significantly reduce albuminuria. Pena et al. [26] aimed to (post hoc) evaluate the therapy response of atrasentan on the 13 urinary metabolites linked to mitochondrial metabolism. These 13 metabolites were previously discovered by Sharma et al. [42] and have been used in the study by Saulnier et al. [28]. As 4 of the 13 urinary metabolites were below the detection limit, they were excluded from the urinary metabolite index, leading to the inclusion of aconitic acid, citric acid, glycolic acid, homovanillic acid, 2-ethyl-3-hydroxypropionate, 3-hydroxy isobutyrate, 3-hydroxy isovalerate, 3-hydroxy propionate, and uracil. After treatment with atrasentan, the index stabilized but declined further with placebo. This study not only offers a means to evaluate treatment but also provides suggestions about the underlying mechanisms of atrasentan, including the prevention of progression of mitochondrial dysfunction [26].

Similar results were found in a study with a small sample size (n = 31) by Mulder et al. [27], who assessed the effect of dapagliflozin on the same 13 urinary metabolites linked to mitochondrial metabolism. Dapagliflozin significantly increased the urinary metabolite index but had no effect on plasma metabolites (measured for the sole purpose of calculating fractional excretion), suggesting a kidney-specific response and supporting the hypothesis that the underlying mechanism is linked to the mitochondrial function of the kidney.

### 3.3. Diabetic Kidney Disease and Plasma/Serum Omics

The blood contains a diverse range of circulating biomolecules that provide a systemic representation of both physiological and pathophysiological mechanisms. Although it is more invasive than collecting urine, it is still easily obtained and a part of routine clinical examination. In addition, it is one of the most biobanked samples that can be used for scientific investigations. Serum was obtained from coagulated blood, which required no additives. Plasma was obtained by mixing the blood with an anticoagulant to inhibit clotting. Depending on the research question, one should carefully consider which blood collection matrix to use [43].

#### 3.3.1. Plasma/Serum Proteomics

##### Plama/Serum Kidney Injury Molecule-1 (KIM-1)

This review withheld five articles on plasma/serum KIM-1 [23,27,35,36,37].

##### Diagnostic and Prognostic Biomarker

In the study of Nowak et al. [13], with non-proteinuric (normoalbuminuric and mi-cro-albuminuric) patients with T1DM and normal kidney function, baseline plasma KIM-1 was linked to early damage to proximal tubules and associated with onset and progression of early renal decline (Odds ratio for doubling of plasma KIM-1 for early renal decline was 1.28 (95% CI: 1.07–1.54) in normoalbuminuric patients to 1.35 (95% CI: 1.15–1.58) in microalbuminuric patients). As it is elevated prior to any detectable change in albuminuria, it can be considered as a possible diagnostic and prognostic biomarker.

Similar results have been reported by Gutierrez et al. [17]. In their post hoc analysis of the REGARDS study, in 594 patients with a history of stroke, DM, and eGFR < 60 mL/min/1.73 m^2^, it was shown that plasma markers of inflammation and tubular injury (plasma KIM-1) were associated with a higher risk of ESKD, independent of baseline eGFR and uACR. The HR of the highest quartile of KIM-1 was 2.51 (95% CI: 0.99–6.35).

The post hoc analysis of the CANVAS trial of Sen et al. [10], including 3523 patients with eGFR > 30 mL/min/1.73 m^2^, concluded that doubling in baseline KIM-1 was associated with higher risk of kidney outcomes, with an HR of 1.5 (95% CI: 1.2–1.8) [24]. However, this finding was not confirmed by Waijer et al. [11]. Post hoc analysis of 2553 patients who were normoalbuminuric in the CANVAS trial did not show a significant association between baseline plasma KIM-1 levels and kidney outcomes. Further analysis and results of this study concerning TNFR-1 and TNFR-2 are discussed later.

Earlier studies analyzed different single omics without combining them or considering them as a panel of markers [11,13,17]. Colombo et al. [15] performed a post hoc study of the SDRNT1BIO cohort, including only patients with T1DM with eGFR > 30 mL/min/1.73 m^2^. They performed univariate analysis of KIM-1 as a single marker and combined KIM-1 with TNFR-1, CD27, α-1-microglobulin, syndecan 1, thrombomodulin, cystatin-C, matrix metalloproteinase-8, and clusterin into a panel. In the univariate analysis, when restricted to patients who were normoalbuminuric or microalbuminuric at baseline, KIM-1 was significantly associated with both final eGFR and progression to eGFR < 30 mL/min/1.73 m^2^. This association was not observed in patients with macroalbuminuria at the baseline. When using the panel, the biomarkers outperformed uACR alone in predicting the final eGFR, with TNFR-1 and KIM-1 containing more than 75% of the information contained in the whole set of biomarkers. 

##### Biomarker of Therapeutic Response 

Only one study in our review evaluated KIM-1 as a therapeutic marker. Sen et al. [10] performed a post hoc analysis on the data of the CANVAS trial, consisting of a large and broad population of patients with T2DM and established cardiovascular disease, to examine the effect of the SGLT2 inhibitor canagliflozin on (amongst others) plasma KIM-1 and assessed whether early changes in the biomarker could predict cardiovascular, kidney, and heart failure outcomes. The initial analysis confirmed an increased risk of kidney outcomes with higher KIM-1 scores at baseline, similar to earlier studies. However, although canagliflozin decreased KIM-1 as compared to placebo (26.7% at year 1, 95% CI: 22.7–30.7), changes in the baseline of KIM-1 were not associated with changes in cardiovascular or kidney outcomes.

##### Tumor Necrosis Factor Receptor 1 and 2

TNFR-1 and TNFR-2 are circulating receptors of the pro-inflammatory cytokine TNF-α, which initiates signaling cascades with a crucial role in apoptosis, immune regulation, and inflammation. TNF pathways have also been implicated in endothelial injury and glomerular damage, which are observed in DKD [17]. This review included five articles that analyzed both TNFR-1 and TNFR-2 [10,11,12,15,17].

##### Diagnostic and Prognostic Biomarker

Several studies have demonstrated the value of TNFR-1 and TNFR-2 as diagnostic and prognostic biomarkers for CKD progression. In a post hoc analysis of the CANVAS trial, including patients with T2DM and normoalbuminuria, Waijer et al. [36] observed that each doubling of baseline TNFR-1 and TNFR-2 was associated with a higher risk of kidney outcome (defined as a composite of 40% reduction in eGFR, sustained eGFR < 15 mL/min/1.73 m^2^, need for kidney replacement therapy, and kidney-related death), with HRs of 4.2 (95% CI: 1.8–9.6) and 2.3 (95% CI: 1.5–3.6), respectively.

This was confirmed by Gutierez et al. [17] in a more racially heterogeneous population; 53% of the participants were black with a lower eGFR at baseline. They stated that TNFR-1 and TNFR-2 were associated with incident kidney failure requiring replacement therapy. HRs per two-fold higher concentration were 2.87 (95% CI: 2.34–3.52) and 5.64 (95% CI: 3.98–8.00), respectively, which remained significant after adjustment for eGFR and uACR. However, findings concerning KIM-1 were not comparable between Waijer [11] and Gutierez et al. [17].

The findings concerning TNFR-2 could not be completely replicated by post hoc analysis of the PRONEDI trial by Fernandez-Juarez [12]. Analysis of 101 patients with T2DM and established DKD, with a median follow-up of 32 months, showed that there was significantly higher all-cause mortality and greater progression of kidney disease in patients in the highest tertile of circulating TNFR-1 than in the other tertiles. Results concerning TNFR-2 showed a similar trend but did not reach statistical significance.

The study by Colombo et al. [15] in participants with T1DM, which was already discussed, looked at both single markers and a panel of markers. TNFR-1, but not TNFR-2, was included in the analysis. The panel analysis has already been described above. In the univariate analyses that looked at associations of single markers with the endpoints, the researchers identified a significantly higher risk (5.80-fold for every standard deviation) of progression to eGFR < 30 mL/min/1.73 m^2^ with higher TNFR-1 values. 

##### Biomarker of Therapeutic Response

The second research question of Fernandez-Juarez et al. [12] was whether RAS blockade had an effect on circulating levels of TNFR-1 and/or TNFR-2. However, this was not the case here. This is an important finding, as it suggests that in patients with advanced DKD (the study included participants with at least moderately decreased kidney function and severe proteinuria), RAS blockade does not modify inflammatory biomarkers and that other interventions might be needed.

In patients with T2DM, established cardiovascular disease, eGFR > 30 mL/min/1.73 m^2^, and micro- or macroalbuminuria, Sen et al. [10] noted that canagliflozin was able to reduce the levels of circulating TNFR-1 and TNFR-2 by 2.8% (95% CI: 3.4–1.3) and 1.9% (95% CI: 3.2–0.2), respectively, leading to a lower risk of kidney outcome at year 1. However, baseline levels of biomarkers cannot predict therapy response.

##### Promarker D

Promarker D is a plasma-based protein biomarker panel discovered through non-targeted omic analysis. It consists of three plasma protein biomarkers, apolipoprotein A-IV (apoA4), CD5 antigen-like (CD5L/AIM), and insulin-like growth factor-binding protein 3 (IGFBP3), measured using mass spectrometry, combined with age, serum high-density lipoprotein (HDL) cholesterol, and eGFR. Promarker D scores were categorized as low-, moderate-, or high-risk. Two studies by Peters et al. [8,9] were included in this review. The first study assessed the prognostic utility of Promarker D regarding future renal function decline, defined as incident CKD (evolution-towards eGFR < 60 mL/min/1.73 m^2^), and eGFR decline > 30% during 4 years of follow-up. After adjusting for treatment effect, participants in the moderate- to high-risk category of Promarker D were at a higher risk for incident CKD (odds ratios 5.29 (95% CI: 4.22–6.64) and 13.52 (95% CI: 10.69–17.11), respectively). Sensitivity ranged from 69.3 to 73.2%, and specificity ranged from 76.7 to 76.8%. The performance of Promarker D in predicting an eGFR decline >30% was poor [9]. The second study examined the predictive ability of Promarker D in terms of therapeutic response and, hence, the association between canagliflozin and the change in Promarker D score. After 3 years of treatment, all participants had lower Promarker D scores as compared to the placebo group; however, this decline was greatest for those classified in the high-risk category (decrease of 5.6% (95% CI: −8.6–−2.5)) [8]. Both studies suggest that Promarker D not only has diagnostic/prognostic ability but might also be able to identify patients who would benefit the most from early intervention [8,9].

##### Other

The majority of single omics that have been examined in the research of Gutierez et al. [17] have already been discussed above. However, three of these factors have not yet been identified. Post hoc analysis of 594 participants with advanced DKD from the RE-GARDS study also examined other markers of inflammation/fibrosis than TNFR-1 and TNFR-2, namely chitinase 3-like 1 (YKL-40), MCP-1, and soluble urokinase-type plasminogen activator receptor (suPAR). YKL-40 was the only marker that remained significantly associated with incident kidney failure, requiring replacement therapy after adjustment for eGFR and uACR. YKL-40 is an indicator of tubular injury severity. To the best of our knowledge, the association between YKL-40 and DKD progression has not been investigated. Pena et al. [44] concluded similar results, and hence a possible link between YKL-40 and progression to renal dysfunction (this study was not included in the general overview due to a non-targeted study design).

Galectin-3 is a β-galactoside-binding protein involved in regulatory functions including immunity and inflammation. It has been found to be associated with glomerular injury and hence, renal fibrosis. Growth differentiation factor 15 (GDF-15) is a cytokine that has been linked to deterioration of kidney function, mortality, and morbidity among patients with T1DM [14,45]. Based on a cross-sectional study of 90 participants with T2DM and any stage of DKD, Hussain et al. [14] showed that galectin-3 was significantly elevated in patients with macroalbuminuria and that GDF-15 was significantly elevated in patients with microalbuminuria and macroalbuminuria. Receiver operating characteristic (ROC) analysis yielded an area under the curve (AUC) of 0.773 (95% CI: 0.677–0.875) for galectin-3 and 0.963 (95% CI: 0.929–0.997) for GDF-15. These results confirmed the potential of both markers in the early detection of DKD. However, additional multi-ethnic, multinational, and multicenter studies are needed.

#### 3.3.2. Plasma/Serum Metabolomics

Although metabolomics is an emerging approach for studying human diseases, research on targeted plasma metabolomics in relation to DKD appears to be poorly represented. In this review, we have included only one study on plasma metabolomics [16]. Multiple studies were screened, and although they provided interesting data on metabolomic signatures and possible pathophysiological pathways, they were excluded mainly because of their non-targeted approach [46,47,48,49].

Zhang et al. [16] explored the relationship between phenylalanine, tryptophan, and tyrosine (aromatic amino acids (AAA)) and DKD in patients with T2DM. A retrospective study of 396 participants classified them into three groups: healthy, T2DM with DKD, and T2DM without DKD. Tyrosine was inversely associated with the DKD stage, presumably due to a decrease in urinary tyrosine excretion (although not determined in this study) and the limited activity of phenylalanine hydroxylase due to increased oxidative stress linked to kidney dysfunction. There was no significant association between DKD and other examined AAA.

## 4. Discussion

DKD is a prevalent microvascular complication of DM and is associated with significantly worse prognosis. Currently used diagnostic markers (eGFR and uACR or albuminuria) lack sensitivity and specificity, particularly in the early stages of the disease. As early intervention can delay or even arrest disease progression, novel biomarkers are needed. Omics, a new field of study involving the comprehensive analysis of various types of biological data at the molecular level, has shown promising results in (early) detection of disease, personalized medicine, therapeutic monitoring, and understanding pathogenesis. Despite promising research findings, these biomarkers have not yet been fully integrated into routine clinical practice. 

The intent of this narrative review was to provide an overview of the currently available data on targeted omics. After an extensive search in both PUBMED and EMBASE, 25 different (panels of) omics were withheld, described, and analyzed within the field of proteomics and metabolomics. Despite the number of studies focusing on other omics, such as genomics, transcriptomics, and lipidomics, in this review, no conclusions can be drawn in these categories. Much of the research is still in the discovery/hypothesis-generating phase, using non-targeted approaches. 

### 4.1. Urinary Omics

Urinary omics outperforms plasma omics in DKD because urine collection is stable and non-invasive. The CKD273 classifier has demonstrated promise in diagnosing early stages of DKD and disease development prior to established markers such as eGFR and uACR. However, its application remains strictly research-based, and more proof is required before adopting it in clinical practice. 

### 4.2. Plasma/Serum Omics

Plasma markers such as KIM-1 and TNFR-1 have been established in studies to have diagnostic and prognostic value in DKD patients. However, their clinical value is still unclear due to differences in study populations and outcome definitions. While these indicators may enhance current diagnostic techniques, their application in routine clinical practice is not now viable without additional validation.

### 4.3. Metabolomics in DKD

While most metabolomic discoveries are still at the exploratory stage and lack direct therapeutic application, the discipline holds great potential for the future management of DKD [50,51]. Metabolomics enable an in-depth study of biochemical changes in disease pathways, particularly those involving oxidative stress, mitochondrial failure, and amino acid metabolism. The accuracy of diagnosing and prognosing DKD may be enhanced by integrating metabolomics into clinical practice, particularly when combined with other omics data and established clinical markers. Subsequent investigations should focus on validating these biomarkers in large, prospective cohorts and developing standardized protocols for their clinical implementation. Additionally, incorporating metabolomic data with machine learning algorithms may facilitate the development of prediction models for DKD progression and therapeutic response, ultimately leading to more individualized and effective treatment plans.

### 4.4. Clinical Implications and Future Directions

The findings suggest that omics-based biomarkers can enhance early identification, risk assessment, and individualized therapy for DKD in clinical settings. Biomarkers such as CKD273, KIM-1, and TNFR-1 demonstrate higher sensitivity and specificity than traditional indicators like eGFR and uACR, especially in early disease stages when interventions are most effective. Current DKD diagnostics, based on eGFR and albuminuria, frequently diagnose the disease after severe kidney damage has occurred. CKD273 is more sensitive and specific than uACR in predicting eGFR decrease to <60 mL/min/1.73 m^2^. TNFR-1 and TNFR-2 are strongly associated with the progression of DKD and adverse renal outcomes, enabling early intervention and better risk stratification as these markers correlate with faster eGFR decline and increased risk of ESKD. Furthermore, proteomic indicators exhibit a higher correlate with eGFR than albuminuria alone, indicating their potential relevance in monitoring therapy responses in DKD patients. Metabolomic profiling also has great potential for early DKD diagnosis and prognosis since it identifies small-molecule metabolic alterations that occur before clinical symptoms. Although the diagnostic advantage of current omics biomarkers over conventional techniques is modest, further research into multi-omics and integrated approaches could significantly improve DKD diagnosis and treatment outcomes. Incorporating these biomarkers into standard clinical practice could enable earlier intervention, more personalized treatment strategies, and potentially better outcomes for patients at high risk of progressing to ESKD. However, technical challenges such as standardization, cost, and validation in larger cohorts must be addressed before metabolomics and other omics can be successfully integrated into routine clinical practice.

## 5. Conclusions

We discussed 25 omics that could serve as potential biomarkers in DKD. However, the full potential of omics in DKD has not yet been completely exploited. However, there is a paucity of data on large longitudinal cohorts. Furthermore, they are expensive, time-consuming, and not yet widely available. Although there is insufficient evidence to support its routine clinical use, current omics research has allowed for a better understanding of the pathophysiology of DKD, and has shown promise in early diagnosis, therapeutic decisions, and prognosis. Future studies should apply good patient selection and consider the integration of different omics data facilitated by machine learning approaches.

## Figures and Tables

**Table 1:** Classification of the described omics.

Urinary Omics		Plasma/Serum Omics	
Proteomics	Metabolomics	Proteomics	Metabolomics
CKD273	Panel of 18 markers ^2^	Promarker D	Tyrosine
eGFR based classifier	Panel of 13 markers ^3^	TNFR-1	Phenylalanine
KIM-1		TNFR-2	Tryptophan
NGAL		Galactin-3	
LFABP		GDF-15	
MCP-1		Panel of 9 markers ^4^	
Angiotensinogen		Chitinase-3-like protein 1	
Cystatin-C		MCP-1	
Panel of 13 markers ^1^		suPAR	
IL-6		KIM-1	

^1^ EGF/MCP-1 ratio, MCP-1, IL-8, EGF, EGF-receptor, IL-18, IL-6, macrophage inflammatory protein-1, amphiregulin, placenta growth factor, IL-4, epiregulin, and heparin-binding EGFR-like growth factor. ^2^ L-asparagine, L-lysine, 4-hydroxyproline, L-proline, L-aspartic acid, L-glutamic acid, L-arginine, L-cysteine, pyroglutamic acid, l-phenylalanine, L-glutamine, norepinephrine, succinic acid, 2-ketoglutaric acid, L-tryptophan, citric acid, and serotonin. ^3^ 2-methyl acetoacetate, 2-ethyl 3OH propionate, 3-hydroxy isovalerate, aconitic acid, citric acid, glycolic acid, 3-hydroxy propionate, 3-methyl cortonyl glycine, 3-hydroxy isobutyrate, homovanillic acid, tiglyglycine, and uracil. ^4^ KIM-1, TNFR-1, cluster of differentiation (CD)27, α-1-microglobulin, syndecan 1, thrombomodulin, cystatin-C, matrix metalloproteinase-8, and clusterin.

**Table 2:** Overview of plasma/serum proteomics and metabolomics.

Name	Type	Omic Type	Diabetes Type	DKD Stage Included	Follow-Up	Sample Size	Diagnostic/ Prognostic	Therapeutic	References
Promarker D	Protein	Multi	T2DM	Microalbuminuria and macroalbuminuria	4 y	3568	Significant	Significant for canagliflozin	[8,9]
TNFR-1	Protein	Single	T2DM	All	32 m–6.2 y	101–3532	Significant	Significant for canagliflozin, not significant for RAS blockade	[10,11,12]
TNFR-2	Protein	Single	T2DM	All	32 m–6.1 y	101–3532	Inconsistent, mostly significant	Significant for canagliflozin, not significant for RAS blockade	[10,11,12]
KIM-1	Protein	Single	T1DM and T2DM	eGFR > 30 mL/min/1.73 m^2^	6.1 y–8 y	462–3532	Inconsistent, mostly significant	Significant (canagliflozin)	[10,11,13]
Galactin-3	Protein	Single	T2DM	All	Cross-sectional	90	Significant	N/A	[14]
GDF-15	Protein	Single	T2DM	All	Cross-sectional	90	Significant	N/A	[14]
Panel of 9 serum markers	Protein	Multi	T1DM	eGFR > 30 mL/min/1.73 m^2^	5.1 y	1629	Significant	N/A	[15]
Tyrosine	Metabolite	Single	T2DM	Unspecified	Cross-sectional	396	Significant	N/A	[16]
YKL-40	Protein	Single	T2DM	eGFR < 60 mL/min/1.73 m^2^, no ESKD	6.2 y	594	Significant	N/A	[17]
MCP-1	Protein	Single	T2DM	eGFR < 60 mL/min/1.73 m^2^, no ESKD	6.2 y	594	Not significant	N/A	[17]
suPAR	Protein	Single	T2DM	eGFR < 60 mL/min/1.73 m^2^, no ESKD	6.2 y	594	Not significant	N/A	[17]
Phenylalanine	Metabolite	Single	T2DM	Unspecified	Cross-sectional	396	Not significant	N/A	[16]
Tryptophan	Metabolite	Single	T2DM	Unspecified	Cross-sectional	396	Not significant	N/A	[16]

**Table 3:** Overview of urinary proteomics and metabolomics.

Name	Type	Omic Type	Diabetes Type	DKD Stage Included	Follow-Up	Sample Size	Diagnostic/ Prognostic	Therapeutic	References
CKD273	Protein	Multi	T1DM and T2DM	All	16 w–19 y	35–1777	Inconsistent, mostly significant, especially in early stages of DKD	Inconsistent (linagliptin, spironolactone, and candesartan)	[18,19,20,21,22,23,24]
eGFR based classifier	Protein	Multi	T2DM	Microalbuminuria	19 y	151	Not significant, but trend	N/A	[20]
Panel of 18 urinary markers	Metabolite	Multi	T2DM	Albuminuria	48 w	145	N/A	Significant (spironolactone)	[25]
A total of 13 urinary metabolites linked to mitochondrial metabolism	Metabolite	Multi	T1DM and T2DM	eGFR > 30 mL/min/1.73 m^2^	6 w–6 y	31–150	Significant	Significant (dapagliflozin, atrasentan)	[26,27,28]
Panel of 13 urinary markers	Metabolite	Multi	T1DM and T2DM	eGFR 20–70 mL/min/1.73 m^2^	2–10 y	1001	Significant	N/A	[29]
KIM-1	Protein	Single	T2DM	All but ESKD at baseline	18 w–8 y	31–1573	Inconsistent	Significant (dapagliflozin)	[13,30,31,32]
NGAL	Protein	Single	T2DM	All but ESKD	18 w–40 m	31–257	Significant	Not significant	[30,32]
LFABP	Protein	Single	T2DM	uACR > 100 mg/g and < 3500 mg/g	18 w	31	N/A	Not significant	[30]
MCP-1	Protein	Single	T2DM	uACR >100 mg/g and < 3500 mg/g	18 w	31	N/A	Not significant	[30]
Cystatin-C	Protein	Single	T2DM	eGFR > 15 mL/min/1.73 m^2^	40.8 m	257	Significant	N/A	[32]
Angiotensinogen	Protein	Single	T2DM	eGFR > 15 mL/min/1.73 m^2^	40.8 m (median)	257	Significant	N/A	[32]
Panel of 13 urinary markers	Protein	Multi	T1DM	eGFR > 30 mL/min/1.73 m^2^	5.1 y	1629	Not significant	N/A	[15]
IL-6	Protein	Single	T2DM	uACR > 100 mg/g and < 3500 mg/g	18 w	31	N/A	Significant	[30]

**Table 4:** Summary of clinical trials investigating CKD273 as a biomarker.

Study Design	Trial	Research Question	Diabetes Type	DKD Stage	Follow Up	Sample Size	Outcome	Conclusion	References
Post hoc	MARLINA T2D	Diagnostic/ prognostic/ therapeutic (Linagliptin)	T2DM	Albuminuria 30–3000 mg/g creatinine	24 w	360	Progressive kidney function loss.	CKD273 can predict kidney outcome and therapy response.	[23]
Prospec-tive	PRIORITY	Diagnostic/ prognostic/ therapeutic (Spironolactone)	T2DM	Normoalbuminuria	2.5 y	1777	Progression to microalbuminuria, impaired kidney function, 30% decrease in eGFR.	CKD273 can predict kidney outcome, but not therapy response.	[19,37,38]
Post hoc		Therapeutic (Spironolactone)	T2DM	All	16 w	111	Change in uACR	CKD273 can predict therapy response.	[24]
Post hoc	DIRECT protect 2	Diagnostic/ prognostic/ therapeutic (Candesartan)	T2DM	Normoalbuminuria	4.1 y	737	Progression to microalbuminuria.	CKD273 can predict kidney outcome, but not therapy response.	[35]
Post hoc	Steno 2	Diagnostic/ prognostic	T2DM	Microalbuminuria	19 y	151	Progression to macroalbuminuria, impaired kidney function, death, ESKD	CKD273 did not predict kidney outcome.	[22]
Post hoc		Diagnostic/ prognostic	T2DM	Microalbuminuria	6 y	155	Mortality and cardiovascular disease. Transition in albuminuria stage.	CKD273 can predict kidney outcome.	[20]
Post hoc		Diagnostic/ prognostic	T1DM & T2DM	Normoalbuminuria	10–15 y	35	Transition in albuminuria stage	CKD273 can predict kidney outcome.	[18]
Post hoc	PREVEND & STENO	Diagnostic/ prognostic	T2DM	Normoalbuminuria and micro-albuminuria	3 y	88	Transition in albuminuria stage	CKD273 can predict kidney outcome.	[21]

**Table 5:** Summary of clinical trials investigating KIM-1 as a biomarker.

Source	Study Design	Trial	Research Question	Diabetes Type	DKD Stage	Follow Up	Sample Size	Outcome	Conclusion	References
Plasma	Post hoc	CANVAS	Diagnostic/ prognostic/ therapeutic (Canagliflozin)	T2DM	eGFR > 30 mL/min/1.73 m^2^	6.1 y	3532	Kidney disease progression (sustained 40% decline in eGFR, ESKD, death related to kidney disease)	KIM-1 can predict kidney outcomes, but not therapy response.	[10]
Plasma	Post hoc	CANVAS	Diagnostic/ prognostic/ therapeutic (Canagliflozin)	T2DM	Normoalbuminuria	6.1 y	2553	Onset of CKD and an eGFR decline of ≥30%	KIM-1 can not predict kidney outcomes and therapy response.	[11]
Plasma	Post hoc	SDRNT1BIO	Diagnostic/ prognostic	T1DM	eGFR > 30 mL/min/1.73 m^2^	5.1 y	1629	eGFR progression to <30 mL/min/1.73 m^2^	The panel including KIM-1 can predict kidney outcomes.	[15]
Plasma	Post hoc	REGARDS	Diagnostic/ prognostic	T2DM	eGFR < 60 mL/min/1.73 m^2^	6.2 y	594	Incident kidney failure needing replacement therapy	KIM-1 can predict kidney outcomes.	[17]
Plasma & Urine	Post hoc	Joslin Kidney	Diagnostic/ prognostic	T1DM	Normoalbuminuria	8 y	462	Early kidney function decline (annual eGFR loss of >3.3%)	KIM-1 can predict kidney outcomes.	[13]
Urine	Post hoc		Therapeutic (Dapagliflozin)	T2DM	uACR > 100 mg/g < 3500 mg/g creatinine, eGFR > 45 mL/min/1.73 m^2^	18 w	31	Change in biomarker	Dapagliflozin reduced urinary KIM-1 excretion.	[30]
Urine	Prospec-tive		Diagnostic/ prognostic	T2DM	eGFR > 15 mL/min/1.73 m^2^	40.8 m	257	Time to development of ESKD or sustained decline in eGFR > 5 mL/min/1.73 m^2^	KIM-1 can predict kidney outcomes.	[32]
Urine	Prospec-tive	FinnDiane	Diagnostic/prognostic	T1DM	all but ESKD	6 y	1573	Transition in albuminuria stage	KIM-1 cannot predict kidney outcomes.	[31]

## Data Availability

Not applicable.

## Abbreviations

eGFR	estimated glomerular filtration rate
GDF-15	growth differentiation factor-15
IL	interleukin
KIM-1	kidney injury molecule-1
LFABP	liver fatty acid binding protein
MCP-1	monocyte chemoattractant protein-1
NGAL	neutrophil gelatinase-associated lipocalin
suPAR	soluble urokinase plasminogen activator receptor
TNFR	tumor necrosis factor receptor
DM	diabetes mellitus
DKD	diabetic kidney disease
ESKD	end-stage kidney disease
N/A	not applicable
RAS	renin–angiontensin system
YKL	tyrosine (Y), lysine (K), and leucine (L) = chitinase-3-like protein 1
uACR	urine albumin–creatinine ratio
IL-6	interleukin-6
